# A Novel Method for Automatic Identification of Breathing State

**DOI:** 10.1038/s41598-018-36454-5

**Published:** 2019-01-14

**Authors:** Jinglong Niu, Maolin Cai, Yan Shi, Shuai Ren, Weiqing Xu, Wei Gao, Zujin Luo, Joseph M. Reinhardt

**Affiliations:** 1 School of Automation Science and Electrical Engineering, Beihang University, Beijing, 100191 China; 2 Department of Respiration, Beijing Anzhen Hospital, Capital Medical University, Beijing, 100029 China; 3 Department of Respiratory and Critical Care Medicine, Beijing Engineering Research Center of Respiratory and Critical Care Medicine, Beijing Institute of Respiratory Medicine, Beijing Chao-Yang Hospital,Capital Medical University, Beijing, 100043 China; 4 Department of Biomedical Engineering, University of Iowa, Iowa City, IA 52246 United States

## Abstract

Sputum deposition blocks the airways of patients and leads to blood oxygen desaturation. Medical staff must periodically check the breathing state of intubated patients. This process increases staff workload. In this paper, we describe a system designed to acquire respiratory sounds from intubated subjects, extract the audio features, and classify these sounds to detect the presence of sputum. Our method uses 13 features extracted from the time-frequency spectrum of the respiratory sounds. To test our system, 220 respiratory sound samples were collected. Half of the samples were collected from patients with sputum present, and the remainder were collected from patients with no sputum present. Testing was performed based on ten-fold cross-validation. In the ten-fold cross-validation experiment, the logistic classifier identified breath sounds with sputum present with a sensitivity of 93.36% and a specificity of 93.36%. The feature extraction and classification methods are useful and reliable for sputum detection. This approach differs from waveform research and can provide a better visualization of sputum conditions. The proposed system can be used in the ICU to inform medical staff when sputum is present in a patient**’**s trachea.

## Introduction

Patients supported by mechanical ventilation are intubated with an endotracheal tube to allow for gas transport to and from the lungs. In critically ill patients, mechanical ventilation can be required to sustain life. However, mechanical ventilation and intubation also cause complications that must be monitored for and managed by medical staff^1,2^. One common condition in intubated patients is the accumulation of excess sputum in the trachea and endotracheal tube. If uncleared, sputum can occlude the airway and lead to hypoventilation and carbon dioxide retention^3,4^, which can affect the therapy and threaten the patient’s life. Therefore, it is necessary to clear the sputum from the trachea in a timely manner.

The sputum clearance is performed by medical staff who check for sputum accumulation via lung sound auscultation. Auscultation is performed by placing a stethoscope on the chest and using it to listen to the respiratory sounds for several breaths. However, it is difficult to develop this skill. Medical staff must be well-trained and sufficiently experienced to detect sputum in the trachea using the sounds heard during auscultation. Moreover, monitoring of sputum accumulation by auscultation increases staff workload, is a subjective process and can be influenced by noise and other environmental factors near the patient’s bedside^5–8^. Therefore, it is important to develop a method to identify sputum conditions more accurately.

In previous research, Yamashita *et al*. focused on the method used to detect the sputum condition and used a sparse representation method to extract features for detection of sputum accumulation using audio samples^9^. They classified the sounds into sounds with sputum present and sounds without sputum present; the average accuracy of classification was only 87% and the data were sourced from only three patients. Therefore, opportunities remain for increased accuracy in identification of the sputum condition. Similar research has also been conducted. For instance, Habukawa *et al*. used breath sound analysis to evaluate asthma^10^. Pinho *et al*. used fractal dimension and box filtering to detect crackle sounds in the thorax^11^. Bahoura *et al*. classified respiratory sounds in normal and wheeze classes using pattern recognition methods^12^. Abbasi *et al*. classified normal and abnormal respiratory sounds based on neural networks and support vector machines^13^. Other researchers focused on developing acquisition methods to collect the sound data. Azarbarzin *et al*. used two microphones to record sound data by placing one on the suprasternal notch of the patient’s trachea (tracheal sound microphone) and hanging the other approximately 20–30 cm from the patient’s head (ambient sound microphone)^14^. Waitman *et al*. used two microphones placed on the anterior chest to record respiratory sound signals^15^. Charleston-Villalobos used an array of 25 sound sensors placed on the subject’s back^16^. Other groups gathered respiratory sound data using an electronic stethoscope^17,18^.

These studies demonstrated many types of respiratory sounds, and different respiratory sounds have individual features. It is difficult to directly apply the same feature set to different types of respiratory sounds. Most research focuses on the waveform of the signal and its mathematical procedures and few studies considered image recognition. The devices used to collect the sound data suffer from the same problems. If the microphone is in direct contact with the skin, it can lead to patient discomfort and the patient’s movements create friction noise that affects the recording results.

In this paper, we describe a new system to detect the presence of sputum in the trachea by analysing the frequency content of respiratory sounds. We describe a new waterproof sound sensor that can be easily connected to an existing endotracheal tube and permits the acquisition of high-quality respiratory sound data. The respiratory sounds are analysed using time-frequency spectra and the features are extracted to characterize the unique sounds produced by sputum in the airway. A logistic classifier is trained to automatically detect the presence of sputum based on the respiratory sounds gathered from a single breath. To test our system, 220 respiratory sound samples were collected from 12 intubated patients, with each sample corresponding to the sounds gathered over one breath. Half of the samples were collected from patients with sputum present, and the other half were collected from patients with no sputum present. In a ten-fold cross-validation experiment, the logistic classifier identified breath sounds with sputum present with a sensitivity of 93.36% and specificity of 93.36%.

## Methods

### Sputum detection system

**Figure 1:**
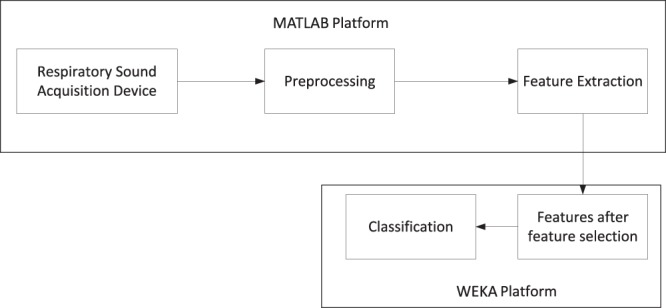
Sputum detection system. Respiratory sound acquisition, preprocessing and feature extraction are performed using custom MATLAB software. Feature selection and classification are performed with the WEKA machine-learning tool.

A block diagram of the system is shown in Fig. 1. The system consists of five major components: (1) respiratory sound data acquisition; (2) preprocessing; (3) feature extraction; (4) feature selection; and (5) classification. Patient respiratory sound data are obtained through the sound acquisition device. Sound segmentation, bandpass filtering, time-frequency transformation, and texture feature enhancement are performed in the preprocessing process. The sputum detection is conducted using feature extraction, feature selection, and machine learning to classify the respiratory sounds.

### Respiratory sound acquisition device

A new respiratory sound acquisition device was developed to record high-quality respiratory sounds free from ambient noise and other artefacts. In our design, the sound sensor is coupled directly to the suction channel of the endotracheal tube. Respiratory sound signals are acquired using a capacitive microphone (SKC MP40 by SKC Acoustics Technology Co Ltd, Beijing, China.) with a frequency response of 20–20,000 Hz and a dynamic range of 30–126 dB. The connector to the endotracheal tube is a T-type device.

**Figure 2:**
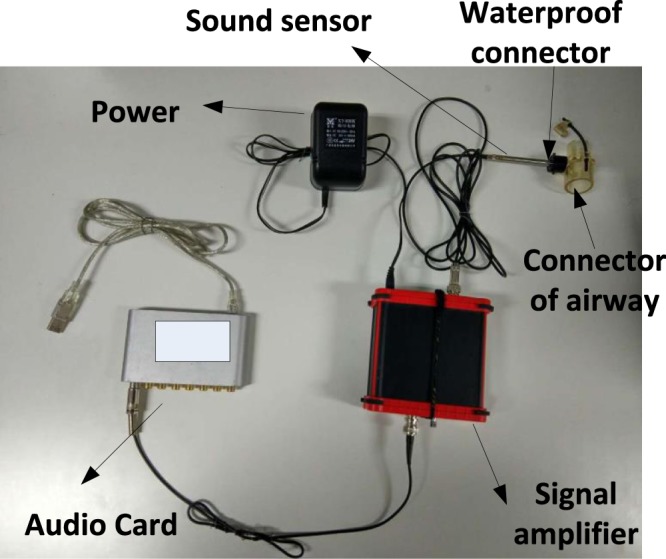
Respiratory sound acquisition device. The device consists of six major components: (1) sound sensor, (2) audio card, (3) endotracheal tube connector, (4) waterproof connector, (5) signal amplifier, and (6) power supply. (b) Prototype acquisition system at the bedside.

A signal amplifier is used to amplify and denoise the sound signal. Because moisture accumulates in the endotracheal tube, waterproofing is used to keep the head of the sound sensor dry. The system can operate for approximately 10–20 minutes without waterproofing. A USB audio card (ESI MAYA 44 by ESI Audiotechnik GmbH, Leonberg, Germany) was used to connect the acquisition hardware with the computer and perform analogue-to-digital conversion. The respiratory sounds were digitized with a single channel at a sampling rate of 44,100 Hz and 16 bits of A/D conversion. According to the frequency analysis, the respiratory sound signal recorded in this paper displays the bulk of its energy in the range of 20 Hz to 6 kHz. Within this frequency range, most of the sound energy from the respiratory tract is below approximately 1000 Hz^19,20^. Therefore, our device can capture almost all of the useful signal. The device is shown in Fig. 2.

### Preprocessing

**Figure 3:**
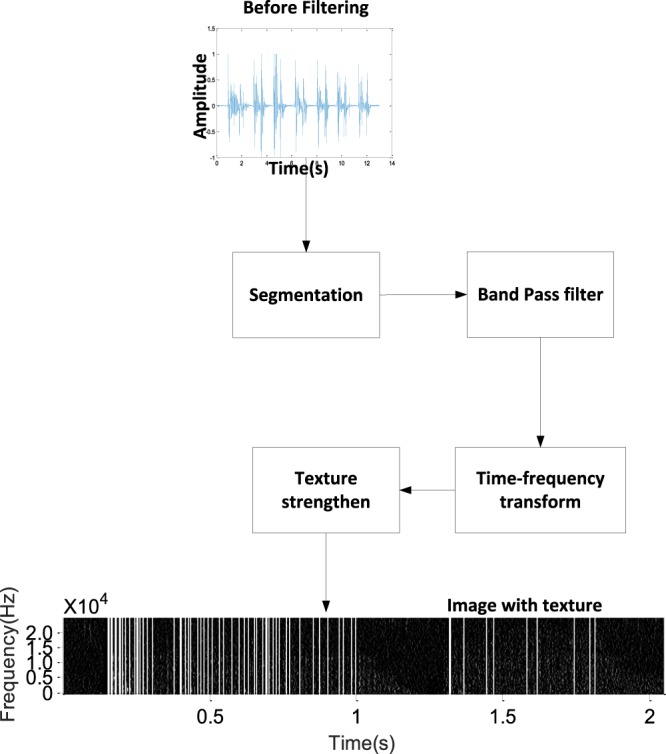
Preprocessing step. Preprocessing divides the sound data into individual breaths, filters the sound samples, and computes and enhances the time-frequency representation of each breath sound.

The preprocessing steps are illustrated in Fig. 3. During preprocessing, the respiratory sound data are separated into individual breaths by detecting the absence of sound between breaths. The individual breath sounds are filtered and transformed into time-frequency data using a short-time Fourier transform. The time-frequency data are enhanced through a texture-strengthening step, as described below. In the preprocessing step, we obtain an image with texture, as shown in Fig. 3. Based on this image, the features of the signal can be extracted.

### Filtering

Due to the design of the respiratory sound acquisition device, the effects of ambient environmental noise are minimized, such as the sounds of people talking. The main sounds acquired by the device are the sputum sound, the sound from the airflow hitting the wall of tube, and the sound of the ventilator. Because the higher frequencies contain some useful information, we focus on the frequency band from 20 to 6000 Hz. In this work, a third-order Butterworth bandpass infinite impulse response(IIR) filter with a bandwidth from 20 to 6000 Hz was used to filter the respiratory sounds prior to additional processing^21^.

### Segmentation of respiratory sound

Sound data are typically acquired from the sensor for one to two minutes, corresponding to several periods of the respiratory cycle. Prior to processing, the data are segmented into components, with each component corresponding to the sounds from a single inhale-exhale cycle. The respiratory cycle endpoint is detected based on the maximum of the autocorrelation function^22^. The autocorrelation is the correlation of the sound signal with a delayed copy of itself. In this paper, the autocorrelation of a set of L sound samples xi, i = 1…L can be calculated as follows:

1 $${R}_{i}(k)=\sum _{m=1}^{L-k}{x}_{i-1}(m){x}_{i}(m+k)$$

where *L* is the length of the data frame and *k* is the time shift used to compute the autocorrelation.

**Figure 4:**
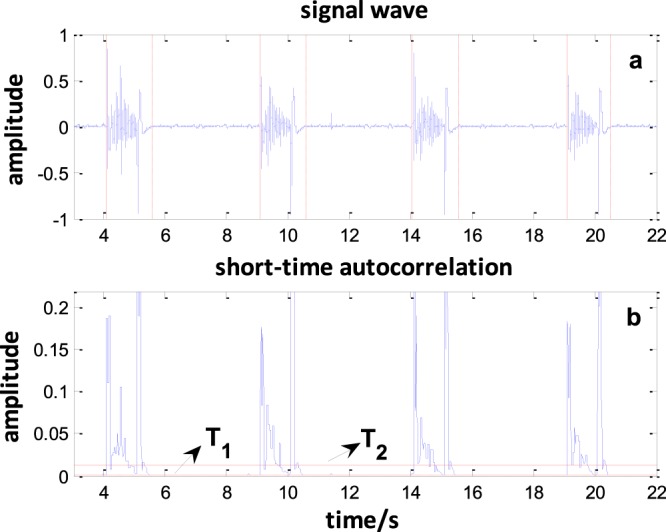
Breath segmentation. (**a**) The original signal; solid red lines mark the start of the cycle and dashed red lines mark the end of the cycle. (**b**) The autocorrelation results. When the value of the short-time autocorrelation of the endpoint is greater than *T*_*2*_ and less than *T*_*1*_, the start and end points of the sound will be obtained. Four respiratory cycles were obtained from this 18-second respiratory signal.

Two thresholds, *T*_1_ and *T*_2_, are used to segment the sound data:

2 $$\begin{array}{c}{T}_{1}=a\,\max (R)\\ {T}_{2}=b\,\max (R)\end{array}$$

where max(*R*) is the maximum autocorrelation calculated in the absence of any breathing sounds, and a and b were experimentally set to 1 and 10 (*a* < *b*). When the maximum autocorrelation is greater than *T*_2_, it can be used to judge the frames of sound belonging to the respiratory cycle. If the maximum autocorrelation is greater or less than the *T*_1_, it can be used to judge the start or end of the respiratory cycle. The segmentation results are shown in Fig. 4. The solid red lines Fig. 4(a) mark the start of the cycle and the short dashed red lines mark the end of the cycle. The autocorrelation results for a set of sound samples are shown in Fig. 4(b).

### Time-frequency transformation

After segmenting the audio sample into individual breaths, the features are extracted from each individual breath audio sample. The feature extraction step has the greatest impact on the ability of a classifier to detect the presence of sputum. In this study, the time-frequency distribution of the audio signal determined from the spectrogram was treated as a 2D image and the texture patterns in this image were analysed to distinguish sputum from non-sputum. The short-time Fourier transform (STFT) is used to obtain the time-frequency distribution of the sound signal^23,24^. The basic concept of the STFT is to introduce a time window that is moved along the signal such that the time-indexed spectrum can be calculated. The STFT of a discrete time signal x(n) can be calculated as

3 $$X(n,k)=\sum _{m=0}^{\infty }x(m)\,\ast \,w(n-m)\,\ast \,\exp [\frac{-2\pi jkm}{N}]$$

where *m* is the number of frame advances of the signal and N is the length of the frame advance of the signal. The function *w*(*n*) is the window function that is zero-valued outside of some chosen interval. In this study, the Blackman-Harris window is used. Computing the result of this equation with a digital signal and a specific window function yields a matrix of complex numbers that can be expressed as follows:

4 $$X(n,k)=\sum _{m=0}^{N-1}{x}_{n}(m){e}^{-j\frac{2\pi km}{N}}$$

The power spectrum of *x* is computed from *X*(*n*, *k*) as

5 $$E(n,k)={|X(n,k)|}^{2}=(X(n,k))\times (conj(X(n,k)))$$

*E*(*n*, *k*) can be interpreted as an intensity image, where *E*(*n*, *k*) represents the pixel intensity values and the indices n and k are treated as the horizontal and vertical axes, respectively. The log transform 10 log_10_(*E*(*n*, *k*)) is used to convert the resulting intensity image to a decibel scale.

### Texture feature enhancement

In this paper, the texture in the time-frequency power spectrum image is used to represent the signal features. The power spectrum can describe a plot of the portion of the signal’s power (energy per unit time) falling within the given frequency band. According to our preresearch^25^, if sputum is present in the respiratory tract, increased high-frequency energy content appears in the audio signal, corresponding to a pronounced vertical texture in the power spectrum image of the sound signal. The noise from the airflow hitting the wall and ventilator consists of white noise, which is represented by a series of points that are evenly distributed in all domains. Therefore, the noise can be treated as the background to show the signal textures in the image. If no sputum is present in the respiratory tract, less vertical texture appears in the power spectrum image. Thus, the presence of high-frequency energy, depicted as vertical texture in the power spectrum, can be used to detect the presence of sputum. Although the noises are treated as the background to show the texture, the energy of the sound with sputum is not continuous at times. Thus, it is desirable to enhance the texture features prior to classification.

Two steps were used to enhance the texture features. The first step detects the edges of the vertical lines in the power spectrum image. A 6 × 6 convolutional mask M was designed as follows:

$$\begin{array}{rcl}M & = & [\begin{array}{llllll}-\,1 & 2 & -\,1 & 2 & -\,1 & -\,1\\ -\,1 & 2 & -\,1 & 2 & -\,1 & -\,1\\ -\,1 & 2 & -\,1 & 2 & -\,1 & -\,1\\ -\,1 & 2 & -\,1 & 2 & -\,1 & -\,1\\ -\,1 & 2 & -\,1 & 2 & -\,1 & -\,1\\ -\,1 & 2 & -\,1 & 2 & -\,1 & -\,1\end{array}]\end{array}$$

The mask M is convolved with the power spectrum image. This operation strengthens the vertical lines and suppresses nonvertical structures^26^. In the second step, the Laplacian of the Gaussian (LoG) operator is used to detect the vertical edges. Because the LoG operator includes a low-pass smoothing step, noise and spurious responses are reduced^27^. The low-pass filtering operation uses a Gaussian kernel, which can be written as:

6 $${G}_{\sigma }(x,y)=\frac{1}{\sqrt{2\pi {\sigma }^{2}}}\exp [-\frac{{x}^{2}+{y}^{2}}{2{\sigma }^{2}}]$$

The convolution between the image and Gaussian function can be written as follows:

7 $${\rm{\Delta }}|{G}_{\sigma }(x,y)\ast f(x,y)|=)|{\rm{\Delta }}{G}_{\sigma }(x,y|\ast f(x,y)=LoG\ast f(x,y)$$

because

8 $$\frac{d}{dt}[h(t)\ast f(t)]=\frac{d}{dt}\int f(\tau )h(t-\tau )d\tau =\int f(\tau )\frac{d}{dt}h(t-\tau )d\tau =f(t)\ast \frac{d}{dt}h(t)$$

The Laplacian of Gaussian *ΔG*_*σ*_(*x, y*) can be obtained and subsequently convolved with the input image. The first and second spatial derivatives of the Gaussian function are written as

9 $$\frac{\partial }{\partial x}{G}_{\sigma }(x,\,y)=\frac{\partial }{\partial x}{e}^{-({x}^{2}+{y}^{2})/2{\sigma }^{2}}=-\,\frac{x}{{\sigma }^{2}}{e}^{-({x}^{2}+{y}^{2})/2{\sigma }^{2}}$$

and

10 $$\frac{{\partial }^{2}}{{\partial }^{2}x}{G}_{\sigma }(x,\,y)=\frac{{x}^{2}}{{\sigma }^{4}}{e}^{-({x}^{2}+{y}^{2})/2{\sigma }^{2}}-\frac{1}{{\sigma }^{2}}{e}^{-({x}^{2}+{y}^{2})/2{\sigma }^{2}}=-\,\frac{{x}^{2}-{\sigma }^{2}}{{\sigma }^{4}}{e}^{-({x}^{2}+{y}^{2})/2{\sigma }^{2}},\,{\rm{respectively}}.$$

The Laplacian of Gaussian (LoG) operator is defined as

11 $$LoG={\rm{\Delta }}{G}_{\sigma }(x,\,y)=\frac{{\partial }^{2}}{{\partial }^{2}x}{G}_{\sigma }(x,\,y)+\frac{{\partial }^{2}}{{\partial }^{2}y}{G}_{\sigma }(x,\,y)=-\,\frac{{x}^{2}+{y}^{2}-2{\sigma }^{2}}{{\sigma }^{4}}{e}^{-({x}^{2}+{y}^{2})/2{\sigma }^{2}}$$

The next step strengthens the vertical texture. After convolution with the LoG operator, a new image matrix is obtained. The vertical textures in the image consist of some line segments. In this work, triple thresholds are used to detect the line segments in the image and extend the segment lines to the whole image. The three thresholds are the zero-crossing rate (ZCR_th_), short-band energy (SBE_th_), and total energy (TE_th_).

The ZCR is the rate of value changes along the elements in the row of the image matrix, i.e., the rate at which the elements in the row change from zero to nonzero or vice versa. The energy is the sum of the squares of the elements in the row of the image matrix and is used to determine whether there are sufficient points to form continuous lines. The SBE is used to detect whether the data in the window size have sufficient energy to form a line. The TE is used to determine whether this time point contains sufficient energy to produce sound. The zero-crossing rate may be low for a continuous line and high for a discontinuous line whereas the energy may be high for a continuous line and low for a discontinuous line. Thus, when ZCR < ZCR_th_, SBE > SBE_th_ and TE > TE_th_, the discontinuous texture is strengthened to be a continuous line.

The ZCR is defined as

12 $${Z}_{n}=\frac{1}{2}\sum _{m=0}^{N}|{\rm{sgn}}[{{\rm{x}}}_{n}({\rm{m}})]-{\rm{sgn}}[{{\rm{x}}}_{n}({\rm{m}}-1)]|w(n-m)$$

where *n* is the column number in the image matrix, m is the row number, and *N* is the length of the row. The zero-crossing rate is used to determine whether the line is continuous.

The SBE is defined as

13 $${E}_{n}=\sum _{m=0}^{N}[x(m)w(n-m){]}^{2}$$

where *w*(*n − m*) is the window function.

The TE is defined as

14 $${E^{\prime} }_{n}=\sum _{m=0}^{N}{x}^{2}(m)$$

### Feature extraction

Grey-level co-occurrence matrices (GLCMs) are used to quantitatively evaluate the textural parameters^28–30^. A GLCM is a matrix in which the number of rows and columns is equal to the number of grey levels G in the image. The matrix element P(i, j| d, θ) is the relative frequency with which two pixels separated by a pixel distance d = $\sqrt{{\rm{\Delta }}{x}^{2}+{\rm{\Delta }}{y}^{2}}$ in the θ direction have grey-level intensity values i and j. The element P(i, j| d, θ) contains the second-order statistical probability and can be written as

15 $$\begin{array}{l}P(i,\,j,\,d,\,\theta )=\{(x,\,y),\,(x+{\rm{\Delta }}x,\,y+{\rm{\Delta }}y)|f(x,\,y)=i;\\ f(x+{\rm{\Delta }}x,\,y+{\rm{\Delta }}y)=j\}\end{array}$$

Due to their large dimensionality, the GLCMs are highly sensitive to the size of the texture samples from which they are estimated. Thus, the number of grey levels is often reduced. Prior to matrix calculation, the input grey level of the image was reduced to 16 levels while maintaining the histogram shape (histogram shapes are used to describe the distribution of texture in an image). Four important additional texture features, i.e., the angular second moment (energy), inertial moment, correlation, and entropy, were computed from the GLCM and used to describe the image texture. These features are defined as follows:

Angular second moment (energy):

16 $$Energy=\sum _{i=1}^{g}\sum _{j=1}^{g}{P}^{2}(i,\,j,\,d,\,\theta )$$

Inertia moment:

17 $$IN=\sum _{i=1}^{g}\sum _{j=1}^{g}[{(i-j)}^{2}{P}^{2}(i,\,j,\,d,\,\theta )]$$

Correlation:

18 $$Correlation=\sum _{i=1}^{g}\sum _{j=1}^{g}[i\times j\times P(i,\,j,\,d,\,\theta )-{u}_{1}\,\times \,{u}_{2}]/({d}_{1}\times {d}_{2})$$

where

$$\begin{array}{l}{u}_{1}=\sum _{i=1}^{g}i\sum _{j=1}^{g}P(i,\,j,\,d,\,\theta ){u}_{2}=\sum _{j=1}^{g}j\sum _{i=1}^{g}P(i,\,j,\,d,\,\theta )\\ {d}_{1}^{2}=\sum _{i=1}^{g}{(i-{u}_{1})}^{2}\sum _{j=1}^{g}P(i,\,j,\,d,\,\theta ){d}_{2}^{2}=\sum _{j=1}^{g}{(j-{u}_{1})}^{2}\sum _{i=1}^{g}P(i,\,j,\,d,\,\theta )\end{array}$$

Entropy:

19 $$Entropy=-\,\sum _{i=1}^{g}\sum _{j=1}^{g}P(i,\,j,\,d,\,\theta )\times {\mathrm{log}}_{10}P(i,\,j,\,d,\,\theta )$$

Each feature measure was obtained for 4 angles (θ = 0°, 45°, 90°, and 135°). Therefore, we obtained 4 × 4 = 16 features.

### Feature selection and classification

Before the classification step, the relevant and descriptive features should be selected. Of the 16 initial features, some might not be sufficiently informative and others might be closely correlated and redundant. To determine the optimal feature set, the information gain selection technique was implemented. The method is used to evaluate the worth of a feature by measuring the information gain with respect to the class^31^. The information gain (IG) for a feature is defined as follows:

20 $$IG=-\,{\sum }_{i=1}^{m}p({c}_{i})\mathrm{log}\,p({c}_{i})+p(t){\sum }_{i=1}^{m}p({c}_{i}|t)\mathrm{log}\,p({c}_{i}|t)$$

where *c*_*i*_ denotes the set of categories, *P*(*c*_*i*_) represents the probability when the result of classification is *c*_*i*_, and *P*(*c*_*i*_*|t*) denotes the probability when the features include t. In this paper, the gain ratio and correlation method were implemented; these methods comprise the filter method. Based on the value rank, the 13 most important features were selected.

The presence of sputum can be recognized by classifying the audio sample based on the selected features. Many possible classification algorithms are available, including neural networks, Bayesnet, regression trees, and linear discriminant analysis^32–34^. Different methods can produce different results in different applications. In this paper, we classify the data into two components: sounds with sputum and sounds without sputum. This is a dichotomous classification problem. The logistic classifier is a popular and efficient method for solving this type of problem. Therefore, the logistic classifier was chosen and implemented in the open source machine-learning software WEKA (University of Waikato, New Zealand, Version 3.8.1).

### Informed consent statement

Data collection was performed by the doctor at the Chao Yang Hospital. All measurements were collected with informed consent from the participants and their relatives. Informed consent signatures were obtained from participants or their legal representatives. The participants and the Chao Yang Hospital ethics committee approved these measurements (Approval Number: 20175241). All measurements were performed in accordance with the relevant guidelines and regulations.

## Results

### Experimental steps

The experimental audio data were recorded in the intensive care unit of Chao Yang Hospital and were collected from two groups. One group was recorded when sputum was present in the respiratory trachea. The other group was recorded after tracheal suction. The first group was denoted as the “with sputum” group and the second group as the “without sputum” group. (The sputum situation was determined by experienced doctors using the auscultation method. In the first step, the doctor used a stethoscope to judge whether sputum was present in the trachea. If the doctor judged that sputum was present in the trachea, recording was started. After recording, sputum clearance was performed. If the sputum was sucked out, the previously recorded data were regarded as data with sputum. After the suction, data were recorded as data without sputum).

In this work, the recordings were collected from intubated patients because the trachea sound recorded is produced when the air flows through the sputum in the trachea. The sound data are related only to the airflow produced by any mode of ventilator. We recorded the breathing sounds from 12 patients. The modes of ventilation used by patients were Pressure Control Ventilation (PCV), Synchronized Intermittent Mandatory Ventilation (SIMV), and Continuous Positive Airway Pressure (CPAP). The data were imported into MATLAB (Mathworks Inc., Natick, MA) for processing. All data were segmented using the maximum of the autocorrelation function. Figure 4 shows the segmentation results. In the preprocessing stage, a Butterworth bandpass filter (20–6000 Hz) was used to remove the redundant information.

**Figure 5:**
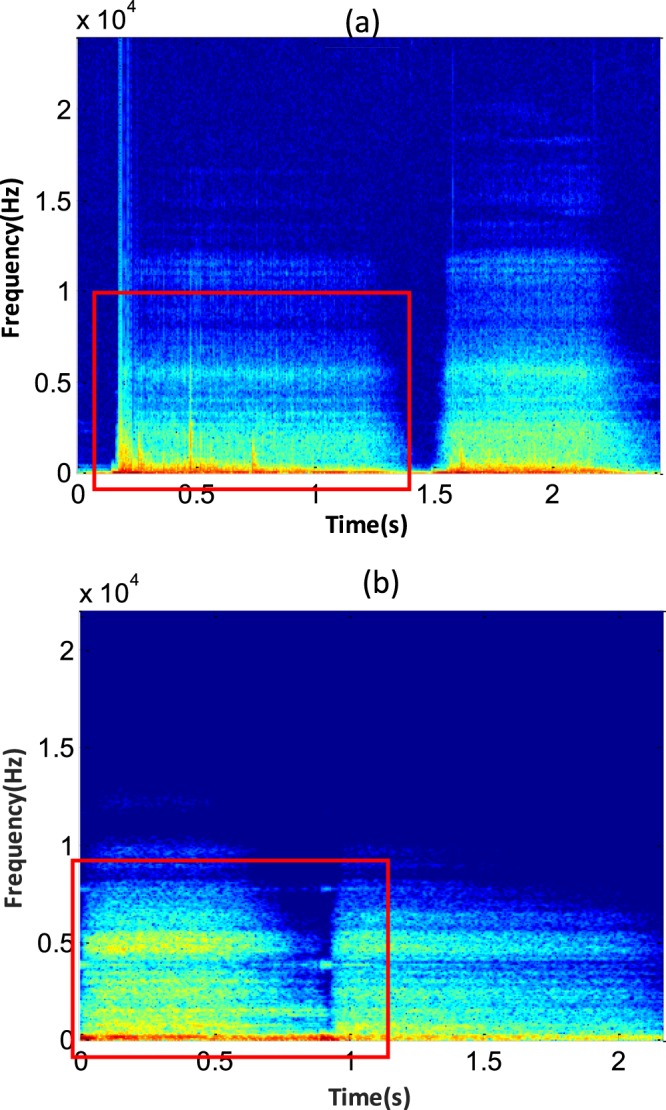
(**a**) Time-frequency distribution of a sound signal with sputum present. (**b**) Time-frequency distribution of a sound signal with no sputum present. The power spectrum with time is displayed on the horizontal axis and frequency is displayed on the vertical axis. The textures can be observed in the red rectangles.

The time-frequency transformation was performed using the STFT. The window size and shift size were set to 1024 and 512, respectively. After obtaining the STFT spectrum, we transformed it into a grey-level image to obtain the grey-level matrix of the image. Figure 5(a,b) show the distribution of the time-frequency of the “with sputum” sound signal and “without sputum” sound signal. The parts with red rectangle show the differences between sound with sputum and sound without sputum, respectively. There are some vertical line textures in Fig. 5(a). In Fig. 5(b), there are very few vertical lines. These features can distinguish audio data with sputum present from that with no sputum present.

**Figure 6:**
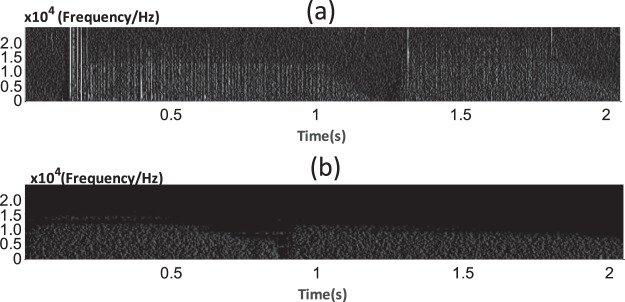
(**a**) Sound signal with sputum present. (**b**) Sound signal with no sputum present. Many more texture segments are observed in the inspiration (0.2–1 s) and expiration (1.5–2 s) phases of the image in (**a**) than that in (**b**).

In the preprocessing steps, texture feature enhancement was applied to obtain the edge of the vertical lines. Figure 6(a,b) can be obtained via the first step of the texture-strengthening method. Figure 6 shows that the spectrum image of the signal is transformed into a grey-level image. With this method, the edge of vertical lines were detected and a certain amount of noise was filtered. There are obvious differences between the images from sound signals with and without sputum.

**Figure 7:**
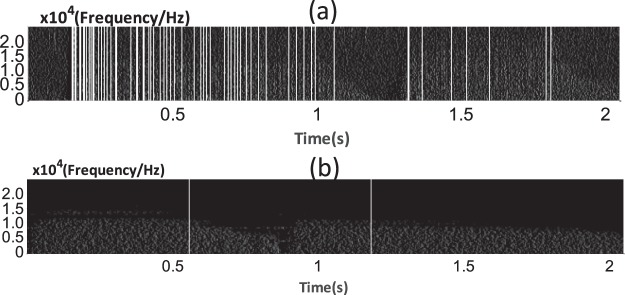
(**a**) Sound signal with sputum present. (**b**) Sound signal with no sputum present. The segments of the lines were extended to full lines based on the proposed method. Several lines are gathered in the inspiration (0.2–1 s) and expiration (1.5–2 s) phases of the sound signal with sputum. For the sound without sputum, there is only one line in inhalation part and only one line in expiration part.

After texture edge detecting, the triple threshold was used to detect the line segments in the image because the texture in the image was typically a discontinuous line segment similar to the image shown above. The aim of this step is to detect the line segments and extend them. Finally, we obtain an image with vertical lines, as shown in Fig. 7. Many vertical lines can be observed in the enhanced spectrum image of a sound signal with sputum. In contrast, few vertical lines appear in the enhanced spectrum image of the sound signal without sputum.

After completing the texture-strengthening steps, the desired features were extracted from the signal using a GLCM.

### Experimental results

Audio data were recorded from 220 instances (110 with sputum, 110 without sputum). The data were preprocessed, and the features were extracted as described above. The information gain, gain ratio, and correlation were calculated and used to evaluate the features. The data are summarized in Table 1. The weight of each feature in the classification is ranked and feature selection is applied according to the ranking. The features shown in bold type in Table 1 are the selected features that optimize the classification accuracy.

**Table 1:** Feature Evaluation Result.

Rank	Correlation		Information Gain		Gain Ratio	
	Feature (direction)	Value	Feature (direction)	Value	Feature (direction)	Value
1	Correlation (90)	0.345	**Correlation (90)**	0.230	Correlation (90)	0.323
2	Inertia (45)	0.329	**Entropy (90)**	0.151	Correlation (45)	0.219
3	Correlation (45)	0.326	**Correlation (45)**	0.148	Correlation (135)	0.214
4	Inertia moment (135)	0.324	**Inertia (45)**	0.148	Entropy (90)	0.198
5	Correlation (135)	0.323	**Inertia (135)**	0.144	Energy (0)	0.196
6	Correlation (0)	0.315	**Correlation (135)**	0.142	Energy (90)	0.196
7	Inertia moment (0)	0.310	**Inertia (0)**	0.140	Energy (135)	0.196
8	Inertia moment (90)	0.138	**Correlation (0)**	0.128	Correlation (0)	0.190
9	Energy (90)	0.098	**Energy (45)**	0.107	Entropy (0)	0.186
10	Energy (45)	0.081	**Energy (0)**	0.105	Entropy (135)	0.186
11	Energy (135)	0.080	**Energy (135)**	0.105	Entropy (45)	0.181
12	Energy (0)	0.076	**Energy(90)**	0.105	Energy(45)	0.169
13	Entropy(135)	0.057	**Entropy (135)**	0.095	Inertia (45)	0.125
14	Entropy (45)	0.056	Entropy (0)	0.095	Inertia (135)	0.122
15	Entropy (0)	0.055	Entropy (45)	0.089	Inertia (0)	0.117
16	Entropy (90)	0.018	Inertia (90)	0.000	Inertia (90)	0.000

**Figure 8:**
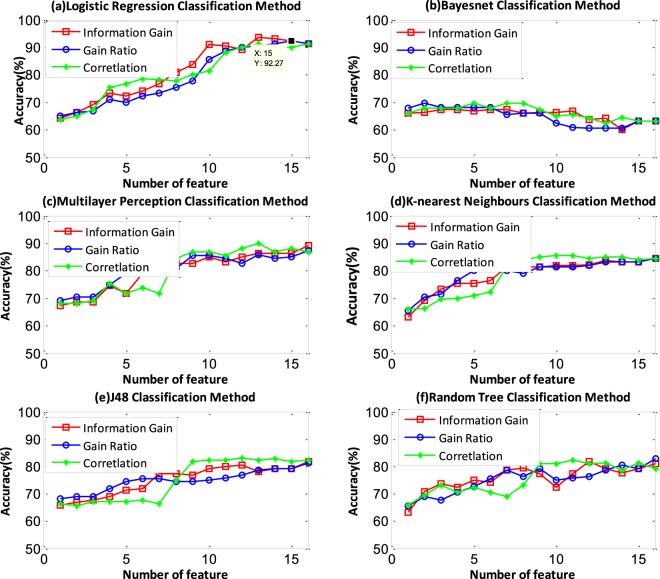
The information gain, gain ratio, and correlation of the feature selection methods are represented by red, blue and green lines, respectively. The logistic classifier shows the best performance and the Bayesnet classifier shows the worst performance. When the logistic classifier is used with the top 13 features ordered based on the information gain, the accuracy reaches 93.63%.

Testing was performed using 10-fold cross-validation. To evaluate the features and the classifier, we compared the performances obtained using different numbers of features and classifiers. Figure 8 shows the classification accuracies using the logistic, Bayesnet, multilayer perception, K-nearest neighbours, J48 and random tree classifiers. The number of features was selected based on the performance, as measured by the information gain, gain ratio, and coefficient of correlation. Each feature set corresponds to a different top number in the results. We obtained 16 feature sets F = {fi |i = 1, 2……, 16}, where i represents the feature number in the feature set. Figure 8 shows that the logistic classifier has better performance than the other methods. The logistic classifier accuracy reaches 93.63% when the information gain technique is used to select the top 13 features. When using the gain ratio and correlation technique to select the features, the highest accuracies reach 92.27% and 91.36%, respectively. The Bayesnet classifier does not work well in this classification problem. The classification accuracy was only 60% and decreases with increasing feature number. Other classifiers have opposite trends. The multilayer perception classifier performs worse than the logistic classifier, but the highest accuracy can reach approximately 89%. The other multilayer perception, KNN, J48, and random tree classifiers show similar performance. The accuracy can reach approximately 80% using the three feature selection methods.

The sensitivity and specificity are introduced to evaluate the performance of the classifiers. The sensitivity measures the proportion of actual positives that are correctly identified and indicates the percentage of sound samples that are correctly identified as sound with sputum. The specificity measures the proportion of actual negatives that are correctly identified and indicates the percentage of sounds samples that are correctly identified as sound without sputum. Table 2 shows the highest accuracy, sensitivity and specificity of classification of each classifier. In this paper, we chose the top 13 of 16 features as the feature vector of the signal based on the ranked list of information gain and chose the logistic classifier as the classification system classifier.

**Table 2:** Highest sensitivity, specificity and accuracy of each classifier.

Classifier	Sensitivity (%)	Specificity (%)	Accuracy (%)
Logistic	93.36	93.36	93.36
Bayesnet	89.09	63.03	69.65
Multilayer Perception	91.51	88.59	90
K-nearest	84.21	86.79	85.45
J48	87.63	79.67	83.18
Random Tree	80.17	83.65	81.82

In addition, two classification experiments were conducted. One classifies the samples without using the texture-strengthening method. The other classifies the samples using the texture-strengthening method. Table 3 shows the confusion matrix for the logistic classifier with 13 features in the two experiments. Compared with the method without a texture-strengthening step, the method with the texture-strengthening step has better classification accuracy.

**Table 3:** Confusion matrix for the logistic classifier with 13 features and the texture-strengthening method

Group	Signal	Classified as		Accuracy (%)
		With sputum	Without sputum	
Without texture-strengthening step	With sputum	94	16	85.5
	Without sputum	15	95	86.4
With texture-strengthening step	With sputum	103	7	93.36
	Without sputum	7	103	93.36

## Discussion

This paper presents a complete system for automatic sputum detection from respiratory sounds and can be part of a method to evaluate patients’ breath state. The implementation was performed in five steps: respiratory data acquisition, preprocessing, feature extraction, feature selection and classification. The data were acquired using a respiratory sound acquisition device. In this device, the sound sensor was installed near the mouthpiece used to connect the ventilator and trachea tubes. This part is universal. Therefore, this structure can be used in intubated patients. Due to the special design, most of the environmental noise can be reduced.

In the preprocessing stage, the maximum of the autocorrelation function was applied to complete the segmentation. The challenge was to select the appropriate threshold value from the first voiceless frames. This method shows good performance in detecting the endpoint of the respiratory sound cycle. If the respiratory rate is too high and the interval between breath cycles is too short, i.e., there is no distinct pause between two breath cycles, the segmentation will fail. However, this situation occurs infrequently or happens when the spontaneous breathing and ventilator are not synchronized. In this situation, the patient and ventilator can have conflicting time steps and the ventilator should be reset.

In the feature extraction processing, the STFT was used to obtain the time-frequency power spectrum image, and the texture in the image will be treated as the features to classify the sounds with or without sputum. Although the noises from the airflow hitting the wall and ventilator are treated as the background to show the texture, the energy of the sound with sputum is not continuous at times. This leads to inaccurate texture feature extraction. Thus, texture strengthening is necessary for feature extraction. The triple-threshold technique was applied to strengthen the texture of the time-frequency power spectrum image. Figure 7 shows that the triple threshold has good performance in strengthening texture. GLCM are used to extract the texture features and the logistic classifier is applied to the texture features to classify the sound samples. Table 3 shows that the sensitivity and specificity increased by 7% and 8%, respectively. The texture-strengthening procedure contributes to increasing the classification accuracy.

Previous research [9] showed that the traditional recording method, an electronic stethoscope, is easily affected by background noise (talking, device operation sounds, and friction sounds between the stethoscope and patient’s skin). Moreover, the stethoscope must be operated manually by a doctor, which is time consuming, and sounds cannot be classified in real-time. The system proposed here can be used to record data without manual operation and allows for real-time classification. Background noise such as talking and device operation can be shielded from the tube. There are many kinds of respiratory sounds. The normal sounds include tracheal breathing sounds, bronchovesicular breath sounds, bronchial breath sounds and vesicular breath sounds. The adventitious sounds include wheeze, rhonchus, sputum sounds, crackle, egophony and pleural friction rub. Some sounds are high-pitched, such as wheeze and fine crackle. Some are low frequency, such as rhonchus or coarse crackle. The accuracy of sound classification depends on its special features. In this study, the textures in the power spectrum image of sound are treated as the features to classify the types of sound. For sounds with higher frequency, the texture direction will tend to be horizontal. For the sounds with lower frequency, the texture direction will tend to be vertical^35^. Therefore, four directions can represent most of the texture directions of a respiratory sound signal. The implementation of the feature extraction method shows that the texture of four directions of information was required to classify the sputum situation.

In terms of classification, in this paper, more classifiers were selected and it was a challenge to decide which method was suitable. WEKA software supplied a flexible platform to test many algorithms, as shown in Fig. 8 and Table 2. The logistic method produced the highest classification accuracy of 93.36%. The confusion matrix shown in Table 3 lists the accuracy of classification, which reached 93.36% and 93.36% for the sounds with sputum present and sounds with no sputum present, respectively.

## Conclusion

A sputum detection system based on texture feature extraction and a logistic classifier is proposed for recognition of sputum sounds. Most research in this area focuses on the waveform of a signal and its mathematical procedures. These methods differ from image recognition, which can offer a better visualization of sputum conditions. Consequently, it is necessary to prepare visual-based evidence of the auscultation sound. This novel method is easier for a doctor to understand. In addition, the classification accuracy is high and can meet the requirements of doctors. The proposed system can be used in the ICU to inform medical staff when sputum is present in a patient’s trachea. Possible improvements to this system include exploring additional features, enhancing the classification system, and extending the system to analyse that multiple breaths to make a classification decision.

## Data Availability

The datasets generated and analysed during the current study are available in the Baidu cloud disk repository, https://pan.baidu.com/s/1Q2O4XhAO6bODMJxH_rwd2Q code: umcx.

## References

[1] Brower RG (2000). Ventilation with lower tidal volumes as compared with traditional tidal volumes for acute lung injury and the acute respiratory distress syndrome. N. Engl. J. Med..

[2] Sutherasan Y, Vargas M, Pelosi P (1997). Protective mechanical ventilation in the non-injured lung: review and meta-analysis. Crit Care..

[3] Lucchini A (2011). Tracheal Secretion Management in the Mechanically Ventilated Patient: Comparison of Standard Assessment and an Acoustic Secretion Detector. Respir Care..

[4] Guglielminotti J, Desmonts JM, Dureuil B (1998). “Effects of Tracheal Suctioning on Respiratory Resistances in Mechanically Ventilated Patients. Chest..

[5] Dasari B (2014). Clinical Examination Skills for Healthcare Professionals. Br J Occup Ther..

[6] Yao, H. D., Ma, J. L. & Dong, M. C. A study of heart sound analysis techniques for embedded-link e-health applications. Presented at *ICIDIT*2*0*1*4*. 87–91 (2014).

[7] Sarkar M, Madabhavi I, Niranjan N, Dogra M (2015). Auscultation of the respiratory system. Ann Thorac Med..

[8] Jones AYM (2014). The effect on sound generation of varying both gas flow rate and the viscosity of sputum-like gel in a simple tubular model. Lung..

[9] Yamashita, T. *et al*. Sparse Representation of Audio Features for Sputum Detection from Lung Sounds. *Presented at ICPR2012 International Conference on*. *IEEE*. 2005–2008 (2012).

[10] Habukawa C (2012). A New Modality Using Breath Sound Analysis to Evaluate the Control Level of Asthma. Allergol Int..

[11] Pinho, C. *et al*. Automatic Crackle Detection Algorithm Based on Fractal Dimension and Box Filtering. *Procedia Comput Sci*. **64** (2015).

[12] Bahoura M (2009). Pattern recognition methods applied to respiratory sounds classification into normal and wheeze classes. Comput Biol Med..

[13] Abbasi, S. *et al*. Classification of normal and abnormal lung sounds using neural network and support vector machines. *Presented at ICEE2013 Iranian Conference on*. *IEEE*. 1–4 (2013).

[14] Azarbarzin A, Moussavi ZMK (2011). Automatic and unsupervised snore sound extraction from respiratory sound signals. IEEE Trans. Biomed. Eng..

[15] Waitman LR (2000). Representation and classification of breath sounds recorded in an intensive care setting using neural networks. J Clin Monit Comput..

[16] Charleston-Villalobos S (2011). Assessment of multichannel lung sounds parameterization for two-class classification in interstitial lung disease patients. Comput Biol Med..

[17] Zolnoori M, Zarandi MHF, Moin M, Teimorian T (2012). Fuzzy rule-based expert system for assessment severity of asthma. J Med Syst..

[18] Riella. RJ, Nohama P, Maia JM (2009). Method for automatic detection of wheezing in lung sounds. Braz J Med Biol Res..

[19] Yadollahi A, Giannouli E, Moussavi Z (2010). Sleep apnea monitoring and diagnosis based on pulse oximetery and tracheal sound signals. Med Biol Eng Comput..

[20] Bohadana A, Izbicki G, Kraman SS (2014). Fundamentals of lung auscultation. N. Engl. J. Med..

[21] Gupta G, Mehra R (2013). Design analysis of IIR filter for power line interference reduction in ECG signals. Int J Eng Res Appl..

[22] Meng M, Ke W, Hong J (2016). Novel DTD and VAD assisted voice detection algorithm for VoIP systems. The Journal of China Universities of Posts and Telecommunications..

[23] Yu SJ (2016). STFT-like time frequency representations of nonstationary signal with arbitrary sampling schemes. Neurocomputing..

[24] Wang WJ, Mcfadden PD (1993). Early detection of gear failure by vibration analysis i. calculation of the time-frequency distribution. Mech Syst Signal Process..

[25] Niu JL (2018). Detection of Sputum by Interpreting the Time-frequency Distribution of Respiratory Sound Signal Using Image Processing Techniques. Bioinfomatics..

[26] Lo WY, Puchalski SM (2008). Digital image processing. Veterinary Radiology & Ultrasound.

[27] Neycenssac, F. Contrast enhancement using the Laplacian-of-a-Gaussian filter. CVGIP. *Graphical models and image processing*. **55** (1993).

[28] Mohanaiah P, Sathyanarayana P, GuruKumar L (2013). Image texture feature extraction using GLCM approach. International Journal of Scientific and Research Publications..

[29] Zucker SW, Terzopoulos E (1980). Finding structure in Co-occurrence matrices for texture analysis. Comput Graph Image Process..

[30] Soh L, Tsatsoulis C (1999). Texture analysis of SAR sea ice imagery using gray level co-occurrence matrices. IEEE Trans. Geosci. Remote Sens..

[31] Fraiwan L (2011). Voiceless Arabic vowels recognition using facial EMG. Med Biol Eng Comput..

[32] Death G, Fabricius KE (2000). Classification and regression trees: a powerful yet simple technique for ecological data analysis. ecology..

[33] Funahashi K (1989). On the approximate realization of continuous mappings by neural networks. Neural Netw..

[34] Li J, Bioucas-Dias JM, Plaza A (2012). Spectral–spatial hyperspectral image segmentation using subspace multinomial logistic regression and Markov random fields. IEEE Transactions on Geoscience and Remote Sensing..

[35] Nogata F (2015). Audio-visual Recognition of Auscultatory Breathing Sounds using Fourier and Wavelet Analyses. Asian Journal of Computer and Information Systems..

